# Early intervention program for very low birth weight preterm infants and their parents: a study protocol

**DOI:** 10.1186/s12887-018-1240-6

**Published:** 2018-08-09

**Authors:** Rita C. Silveira, Eliane Wagner Mendes, Rubia Nascimento Fuentefria, Nadia Cristina Valentini, Renato S. Procianoy

**Affiliations:** 1 Universidade Federal do Rio Grande do Sul, Rua Silva Jardim 1155 # 701, Porto Alegre, RS 90450071 Brazil; 2 Hospital de Clinicas de Porto Alegre, Rua Silva Jardim 1155 # 701, Porto Alegre, RS 90450071 Brazil

**Keywords:** Preterm, Neurodevelopment, Early intervention, parent’s program, Very low birth weight infants, Massage therapy by the mother, Skin-to-skin care

## Abstract

**Background:**

Preterm infants are high risk for delayed neurodevelopment. The main goal is to develop a program of early intervention for very preterm infants that allows families to apply it continuously at home, and quantify the results of early parental stimulation on improvement of cognition and motor skills.

**Methods:**

Randomized clinical Trial including inborn preterm infants with gestational age less than 32 weeks or birth weight less than 1500 g at 48 h after birth. Eligible for begin the intervention up to 7 days after birth. Study Protocol approved by the Brazilian national Committee of ethics in Research and by the institutional ethics committee.

Intervention group (IG): skin-to skin care by mother (kangaroo care) plus tactile-kinesthetic stimulation by mothers from randomization until hospital discharge when they receive a program of early intervention with 10 parents’ orientation and a total of 10 home visits independently of the standard evaluation and care that will be performed. Systematic early intervention program will be according to developmental milestones, anticipating in a month evolutionary step acquisition of motor and / or cognitive expected for corrected age. Active comparator with a Conventional Group (CG): standard care according to the routine care of the NICU and their needs in the follow up program. Neurodevelopment outcome with blinded evaluations in both groups between 12 and 18 months by Bayley Scales of Infant and Toddler Development third edition and Alberta Motor Infant scale will be performed. All evaluations will be conducted in the presence of parents or caregivers in a safe room for the child move around during the evaluation.

**Discussion:**

If we can show that a continuous and global early intervention at home performed by low income families is better than the standard care for very preterm infants, this kind of program may be applied elsewhere in the world. We received grants by Bill and Melinda Gates Foundation, DECIT, Cnpq and Health Ministry. Grand Challenges Brazil: All Children Thriving.

**Trial registration:**

The study was restrospectively registered in ClinicalTrials.gov. in July 15 2016 (NCT02835612).

## Background

Born prematurely and its consequences cause major impact on society and health indicators of population. According to the 2012 “Born Too Soon: The Global Action Report on Preterm Birth” of the World Health Organization, Brazil is ranked 10th among the countries with the highest number of preterm live births and 16th in deaths due to complications of prematurity [1]. The data from 2012 indicate that approximately 3 million babies are born in Brazil each year, 350,000 of whom are born with less than 37 weeks of gestation, and prematurity index is higher in the last three years, including preterm very low birth weight infants (gestational age less than 32 weeks and birth weight less than 1500 g). More than half of the preterm infants with birth weight less than 1500 g and gestational age GA of 23–33 weeks born in public university centers of Brazilian Neonatal Research Network died or were discharged with severe pulmonary, neurological or ophthalmological complications [1, 2].

Assistance in the delivery room with effective resuscitation is important for high risk infants and among the survivors is imperatives a continuous follow up clinic program. Follow up program is a continuous of neonatal and perinatal care and should provide conditions to monitor growth, development and common morbidities with a multidisciplinary team able to fully assess the child and the caregivers, parents, all family and school [3, 4]. According Cochrane Review there is a great deal of heterogeneity between studies due to the variety of early intervention programs and gestational ages included in these studies [5].

Despite of those evidences, the role of the family applying those programs at home is not well studied especially in social deprived environments. It is possible that a care process modified by households is beneficial for neurodevelopment outcome, in both; cognitive and motor skills.

Any early intervention for high risk preterm infant must focus in the parents-infant relationship, environment and behavior attitudes in order to reduce stress among parents of prematurely born children and improves neurovelopment outcome their children during childhood [6]. So, our main hypotheses is that a continuous global early stimulation done by parents at home for very preterm infants is better than the traditional one, and it can be offered to many very preterm infants even in poor environments.

To develop a program of early intervention for very preterm infants that allows families to apply it continuously at home is the main goal. An additional objective of this study protocol is to quantify the results of early stimulation on improvement of cognition and motor skills.

There are several intervention programs involving multisensory and motor stimulations such as, gym, auditory, visual, vestibular and tactile stimulations [7–9]. Developing countries must allocate their resources according to their conditions. Preterm infants require several different professionals to take care of them, and many places do not have enough people to take care of all preterm infants.

` We propose a randomized clinical trial to evaluate a continuous program of early intervention involving very preterm infants’ families in their first 12 to 18 months of life taking the chance of their neuronal plasticity during this period.

## Methods

The study has been designed in accordance with the SPIRIT 2013 statement and we are still collecting data and recruiting the patients. The study setting is Hospital de Clinicas de Porto Alegre, Brazil. Hospital de Clinicas de Porto Alegre, an academic hospital, has more than 3800 deliveries /year and the NICU has 120 to 150 very preterm infants admitted yearly. Obstetric Unit of the hospital has 150 deliveries with gestational age ≤ 32 weeks per year. There is a 20 bed level III Neonatal Intensive Care Unit with conventional and high frequency ventilation, nitric oxide therapy, bed cranial and cardiac echocardiography available any time during the day, 3 on call board certified Neonatologists the whole day. There is also a team available for birth assistance anytime during the day. All included preterm infants and their parents will be followed during neonatal period. There is a follow up clinic that take care of preterm infants with gestational age less than 32 weeks or those with birth weight less than 1500 g birth weight independently of their gestational age cared at our NICU evaluating periodically their growth and neurodevelopment outcomes and forwarding to specialized professionals according to their necessity.

Eligibility criteria: Randomized Clinical Trial including inborn preterm infants with gestational age less than 32 weeks or birth weight less than 1500 g when they complete 48 h after birth. Our inclusion criteria are based in a critical cut off point of gestational age and birth weight according different studies [3, 4, 10, 11]. We decided to approach our study in a group of preterm infants with gestational age less than 32 weeks or those with birth weight less than 1500 g independently of their gestational age. Exclusion criteria: neonates that death prior 48 h after birth with major congenital malformations or inborn errors of metabolism, STORCH complex infections, HIV, or autoimmune conditions.

The study was restrospectively registered in ClinicalTrials.gov. in July 2016 and the first participant was enrolled in the study in February 2016. Protocol record 150,606 has been reviewed and public available on ClinicalTrials.gov. Identifier: NCT02835612.

Interventions: Written informed consent was signed by the parents of all preterm infants that filled inclusion criteria in the study protocol. When they complete 48 h of life they are sequentially randomized. The intervention is planned to start on the 7th day of life according the randomization in two arms:

Conventional group (CG): Standard care, according to the routine care of the NICU: skin-to skin care by mother, kangaroo care, and breast feeding policy. After discharge they are referral for a traditional follow up clinic taking care of the demands according to their necessity; with motor, and cognition evaluations and interventions according to their needs.

Intervention group (IG): Skin-to skin care by mother, kangaroo care, breastfeeding policy plus massage therapy are made by the mothers until hospital discharge. After discharge, they receive standard follow up care plus orientation for a continuous global simulation at home. Early intervention will be according to developmental milestones, anticipating in one month evolutionary step acquisition of motor and / or cognitive expected for corrected age. Besides that, we have a total of 10 home visits promoting guidance and supervision sessions. After each appointment, there is one home visit. A number of ten orientations appointments and ten home visits must be completed for each subject included in the IG. A complete description for each group is detailed below:

Conventional group: Our NICU has worked with Kangaroo mother care (KMC) as the practice of skin-to-skin contact since 1990 for all very low birth weight infants that parents consent to participate and their infants have clinical conditions. We have guidelines including staff knowledge and adequate training with available information to assist and support in developing best-practice guidelines and protocols for implementation this practice. During KMC, the infant, clad in a diaper and cap, is held in an upright prone position against the bare chest of the parent (most often the mother) and covered with clothing and/or a blanket. The duration of skin-to-skin contact is usually one hour per session, with cardiorespiratory and temperature monitoring of the infant during all this time. Although most often provided for stable preterm infants who do not require assisted ventilation, we have offered to preterm infants as young as 26 weeks’ gestational age and birth weight 600 g or more who require respiratory support. We have promoted exclusive breastfeeding. Infants have been discharged home regardless of weight as soon as their mother understood how to care for and feed her infant [12].

In developing countries, KMC for preterm infants has been shown to reduce mortality, severe illness, infection, length of hospital stay and improved mother-infant attachment [13].

After discharge all preterm infants (birth weight < 1500 g) born at the Hospital de Clínicas de Porto Alegre (HCPA) are routinely referred to the Neonatology outpatient clinic for monthly follow-up visits until 6 months of corrected age (CA), bimonthly from 7 up to 12 months corrected age, and every 3 months thereafter until age 24 months, according to routine hospital practice.

A multidisciplinary team is required to form a follow up clinic [3, 4, 10]. Our follow up clinic is coordinated by a neonatologist that understands the infant as a whole. The follow up multidisciplinary team is presented in Table 1.

**Table 1:** Multidisciplinary team in the follow up program

Member of the team	Role in the team
Pediatrician/Neonatologist	Coordination, evaluate growth and screening of development, take care of the general clinical medical problems
Psychologist	Evaluate neurodevelopment using scales, psychological problems, parental infant bonding
Pediatric Neurologist	Manage seizures, cerebral palsy, swallowing problems
Ophthalmologist	Evaluation of Retinopathy of Prematurity (ROP), visual acuity, strabismus
Ear, nose and throat doctor	Evaluation and management of hearing problems
Nutritionist	Management of growth failure
Speech Therapist	Speech problems and swallowing problems
Nurse	Immunization and Hygiene control

During the appointments, feeding orientations, weight gain, general health/illness and infections are recorded. A 24 h dietary recall is administered at each visit to assess feeding routines, use of formula, breast milk, and family food preferences. Routinely feeding orientations have been offered according their needs (exclusive breastfeeding or mixed feeding or infant formula; nutritional requirements according their corrected age). Recurrent admissions during the first year of life, anthropometric parameters (weight, head circumference, body mass index, and length at 6, 12 and 18 months of corrected age (z scores plotted on WHO growth charts), and results of routine tests performed at 1 year of corrected of age are systematically recorded.

Patients will have their motor neurodevelopment evaluated by AIMS (Alberta Infant Motor Scale at 6 and 12 moths of CA) and forwarding to specialized professionals according to their necessity. The scale was translated and adapted to the Portuguese language, being quick and easy to application. The reliability and reproducibility showed satisfactory values [14]. Applications of the Bayley Scales, cognition, language and motor (BSDI- III, Bayley Scale Development Index, version III), by qualified and trained professional will be performed at 12 months CA for all infants [15].

The examiner is able to distinguish, by means of comprehensive and standardized protocols for neurological development, developmental scales and tests between the normal biological variation and the deviant development. The clinical approach has the great advantage of being easily repeated, obtaining developmental trajectories that can lead to neurological disorders being suspected in both arms [16] .

**Intervention group (IG):** We have implemented a program of early, continuous and global intervention with parents’ orientation independently of the standard evaluation and care that will be performed for preterm infants. In NCIU eligible preterms receive skin-to skin care by mother, kangaroo care, breast feeding policy plus the tactile-kinesthetic stimulation by mothers from randomization and after preterm’s are clinically stable (7 days after birth) until hospital discharge. Intervention performed exclusively by the mothers is based on studies regarding the application of skin stimulations and passive exercises in preterm infants [9, 17, 18].

Previously we performed the same early intervention during NICU stay as follow [19]: Mothers are taught to perform a tactile and kinesthetic stimulation four times a day with an interval of 6 h during 15 min. The tactile stimulation is done with two or three fingers with a gently pressure three times in one direction and in the opposite direction on the temporal, frontal, periorbital, nasal and perilabial regions of the face; the external side of the upper and lower limbs. The kinesthetic stimulation was performed with passive [19]. Mothers of the IG are instructed to observe the newborns’ tolerance signs to avoid excessive stimulations.

During NICU stay researchers of our team have had regular meetings with mothers included in IG every 48 h to assure that they are doing the intervention as instructed and to check the parental bond [20]. In previous publication we demonstrated that massage therapy by mothers combined to skin-to-skin care during neonatal hospital stay improved neurodevelopment outcome at 2 years corrected age [21] .

After discharge, preterm infants have standard care of a traditional follow up clinic taking care of the demands according to their necessities and they receive orientation for a continuous global simulation at home besides the usual appointments to the follow up clinic, monthly in the first semester corrected age and bimonthly in the second semester until 12 months corrected age and every 3 months thereafter until age 24 months corrected age. Our study protocol has offered ten additional appointments for systematic orientation for a continuous global simulation at home; and ten home visits during the first 18 months corrected age.

Follow up appointments, home visits, intervention during follow up program and all systematic orientation for early intervention have been done according to developmental milestones, anticipating in a month evolutionary step acquisition of motor and/or cognitive expected for corrected age. The theoretical neurodevelopmentalist referential of the main periods for the acquisition of developmental landmarks have been used [22].

During the sessions the patient should be well fed, have slept his nap and comfortable. Parents are learning to read your preterm’s behavior and respect their needs. We have distributed flyers with techniques and science-based activities to be applied systematically and sequentially at home. Parents have one flyer per appointment; a total of 10 flyers with orientations will be distributed for each subject of IG. Each patient will have a complete book of orientations in the end of the study.

In IG, during first six months corrected age, mother, father and / or corresponding caregivers have been received six flyers: simple guidelines to encourage large motor skills, fine and cognition and some toys.

From discharge up to three months corrected age: Sitting next to their children parents must place them on their stomach on the floor, being sure that their face, mouth, and nose is not covered, using a foam roller to position. Although tummy time is very important, preterm infants should also have time playing on their backs; so stimulation is also performed with the child in the position lying in bed or prone position and/or during bath; a number of three detailed guideline orientations is offered for each included family. Folders with detailed illustrations are offered to parents:

Gross and fine motor stimulation: Crossing arms and relaxation movements (play in the bath for 5 to 10 min, beating hands and feet in water, rubber animals). To make gymnastic movements with flexion and passive extension of the upper and lower limbs; in order to support themselves on the upper limbs in the prone position and to acquire the expected rolling ability.

Cognitive stimulation: Getting close and speak slowly, singing low. Use a mobile to look up, black and white gloves to put in the hands’ mother to play and the child turn your head 180 °C.

Material’s kit for the first, second and third appointments: foam roller to position, rubber animals (3 each/child), black and white woolen gloves, one for each mother / parent intervention group; colorful rattle without light and colored mobile.

From 3 up to 6 months corrected age: a number of three detailed guidelines have been offered to mother/father to stimulate their preterm infant presenting different opportunities to explore, develop skills and abilities in a natural way.

Gross and fine motor stimulation: parents are advised to put back the child to a large plastic ball holding child’s thighs them. Roll the ball slowly forward and back, side to side in order to prepare for the sitting position and obtain equilibrium. To raise the child lying on his back with a nearby color mobile tummy, arms open and extended by the mother holding the child’s hands, showing him that the child should try to catch with their feet; learning to ride. Teach touch objects (sponge rough on one side and the other foam) with different textures (soft, hard, and rough), describing the characteristic touch (stimulates cognition).

Cognitive stimulation: listening to music, singing and reading simple words and short sentences. After the bath, while dry parts of the body with a towel; the mother is guided to speak the body parts that she is drying up; mother says: the foot, the hand, arm and so on…speaking slowly with the child. In the mirror, she shows and names: eyes, mouth, nose, both mother and child, for it to become aware of their individuality. Social interaction is emerging in this ages and need to be stimulated.

Material’s kit for the fourth, fifth and sixth appointments: Colorful rattle with light, plastic large ball, small unbreakable mirror, books with stories, rag doll or cloth toy. Eco sponges commercial home kitchen wash dishes.

From 7 up to 12 months corrected age: study protocol has two detailed guidelines and appointments. Simple guidance for parents has been strengthened in this phase: “Help your child learn to locate things by listening: show her the toy, then put it behind your back and activate the sounds. Do this several times to see if she will crawl to you to find the source of the sound”.

Gross and fine motor stimulation: gymnastics intends to tone the muscles in order to prepare for the first sitting position and then standing without support, to walk independent. Using a large plastic ball (the ball should not be too full) the mother is oriented to hold the child against him, one hand holding his knees thereof and the other the chest. Using the floor with EVA material for the child to have displacement space, the parents play small colored balls twice a week talking about the game (action/reaction). To stimulate fine motor, is guided to offer magazines to be torn by the child, showing how to do it quietly. Once torn into several pieces, teach to make a paper ball with the pieces and play ball with the child.

Cognitive stimulation: In the mirror, mother shows different colored pieces (balls, books) talking slowly each color and numbers, using gestures when she talks to child, linking her actions to her words**.**

From 8 months of corrected age, display and nominate the body parts: head, belly, hand, foot, mouth, nose, eyes. Read books and show the animal pictures, repeating the name of each figure well paused and with the mouth wide open. Material kit for the seventh and eighthappointments: Small colored balls of non-toxic rubber (number = 6). EVA Material (100 cm × 100 cm).Toy fit, action/reaction toy, colored cubes with all geometric forms (one for each subject included in IG).

From 13 up to 18 months corrected age: study protocol has two detailed guidelines and appointments. Parents have been learned to speak and to teach the child mimics what other people do, babbling syllables like words, demonstrating what she wants with gestures; they need to encourage the child to get physically involved with the toy to strengthen muscles and confidence.

Gross and fine motor stimulation: reinforced by the researchers that the game and the play are the best way to stimulate a child. Using a large plastic ball, the mother/father is oriented to tilt the child’s chest to the ball until he/she can put the hands to make a little effort to get up; following the stimulation, the child learns to roll the ball, causing the release of both hands. It is important to advise the mother to release the child gradually hip.

Cognitive stimulation: parents are advised to read a book every day, and when they read these books, talk to child about these feelings. Get him to show how his face looks when he is mad, afraid, etc.… Talk repeatedly reinforcing each child’s achievement is very important all the time.

Material’s kit for the ninth and tenth appointments: Wooden poles with 60 cm in size each, two for each subject in the intervention group; four books with drawings of animals and objects and one case with 12 crayon colors for each subject.

All these activities must not use more than 15 min and they must seem games. Three times / week (alternating with gross and fine motor stimulation) and daily cognitive stimulation are recommended for IG plus conventional care according their needs. All parents will receive the same material kit for stimulation use that is sponsored by Bill and Melinda Gates Foundation. We have be done systematic orientations for cognitive stimulation, fine and gross motor, totaling 10 appointments and 10 home visits promoting guidance and supervision sessions. Systematic orientations will be delivered to parents in all medical appointments at follow up clinic. In the explanatory material for parents each description refers to an activity is a representative figure of the same proposal activity in order clearly to communicate for parents.

The aim of home visits is to evaluate the comprehension of the orientation and to be sure that the intervention has been done by families, a strategy to improve adherence to intervention protocol. During home visits pictures are taken, there is an explanation of the clinical relevance of the study and questions are promptly answered by the multidisciplinary team.

There is a multidisciplinary team involved in the whole study and we will have a critical view of the intervention impact (final evaluation) in both; conventional and intervention groups (Table 1).

### Outcomes

Primary outcome: a global neurodevelopment evaluation will be obtained at 12 to 18 months corrected age for all patients to compare the effect of early systematic intervention independently of formal enriched environments in motor and cognitive aspects. The infants will be evaluated in relation to their motor, and cognitive neurodevelopment using AIMS and Bayley III scales between 12 and 18 months corrected age [14–16].

AIMS (Alberta motor infant scale): a blinded physiotherapist will evaluate the children of both groups between 12 and 18 months with Alberta Infant Motor (AIMS) scale in all eligible patients. The evaluation will be conducted in the presence of parents or caregivers in a safe surface with room for the child move around during the evaluation. The examiner will interact with the child to encourage response, but physical facilitation of movement should be avoided. During the evaluation, they are punctuated behaviors more or less mature within the motor repertoire of the child in each position (supine, prone, sitting and standing). This repertoire is called “motor” window. All items priced within the window motor and the window motor to the previous items are scored. The evaluation of the end, the child will receive a score based on the sum of the items scored on each posture, called raw score. This score will be observed in a standardized chart to find the baby development percentile according to the chronological age or corrected. Percentiles instrument standards are: 5%, 10%, 25%, 50%, 75%. According to this percentile baby’s development can be classified into three categories: normal or typical (percentile> 25%), suspicious (percentile> 5% and ≤ 25%), abnormal or atypical (percentil≤5%) [14].

BSDI-III: *Bayley scales of infant and toddler development third edition*: The Bayley Scales of Infant and Toddler Development, Third Edition, will be used for assessment of neurodevelopment at 12 and 18 months’ corrected age. The scales will be administered at the hospital clinic, on the same day of each follow-up visit, by a psychologist who was blinded to group allocation. Cognitive, motor, and language development will be considered normal if higher than 89; below average if 80 to 89; borderline if 70 to 79; and extremely low if less or equal 69. Examine all the facets of a young child’s development according manual [15].

AIMS and Bayley Scales are recommended to use together and in different ages because false positives are common and therefore it is beneficial to follow-up children at high risk of motor impairment at more than one time point, or to use a combination of assessment tools [8].

Secondary outcome: We have evaluated parental stress and parental infant bonding at hospital discharge in all preterm infants included in the study and survival in the neonatal period.

PARENTAL BONDING INSTRUMENT (PBI) had been applied by a professional blind to the group to which the child belongs. These questionnaires are to be used for research purposes only. PBI is a self-administered Likert scale (0 to 3) instrument, with 25 questions related to father and mother, in which subjects answer how similar those behaviors were to their parents’ behavior until the age of 16 years. The instrument measures two constructs: the first one is affection, which is more consistent and clearly bipolar (affection, heat, availability, care, sensitiveness versus coldness and rejection); the second construct is control or protection (control, intrusion versus encouragement of autonomy) [20].

Emotional availability scales –EAS: this scale will be used to assess both groups at the end of the study. The EAS consists of six scales; four scales assess adult emotions and behavior related to sensitivity, structuring, non-intrusiveness, and non-hostility. The other two scales are related to child behavior. Child responsiveness to the caregiver assess the child interactions with the adult; and child involvement with the caregiver scale assesses the behaviors regarding to child invitations to caregiver to join her in the play and the interaction talks with the caregiver. The scales scores are obtained by scores measured in each dimension using a Likert-type continuous scale with scores between 1 and 7.

Other outcomes: Nutritional conditions during hospital stay and follow up program. Prevalence rates of exclusive breastfeeding and mixed feeding at 6 months corrected age will be recorded to measure maternal bond, in both groups. Anthropometric measures are plotted according to gender and corrected age using the WHO curves. Growth velocity has been registered in the reference curves, using two computers with an appropriate statistical program for nutritional assessment (ANTHRO). There is a nutritionist of the multidisciplinary group that makes home visits and other nutritionist to perform the growth evaluations in the exact moments in each group (blinded to which group the child was previously allocated).

**Fig. 1:**
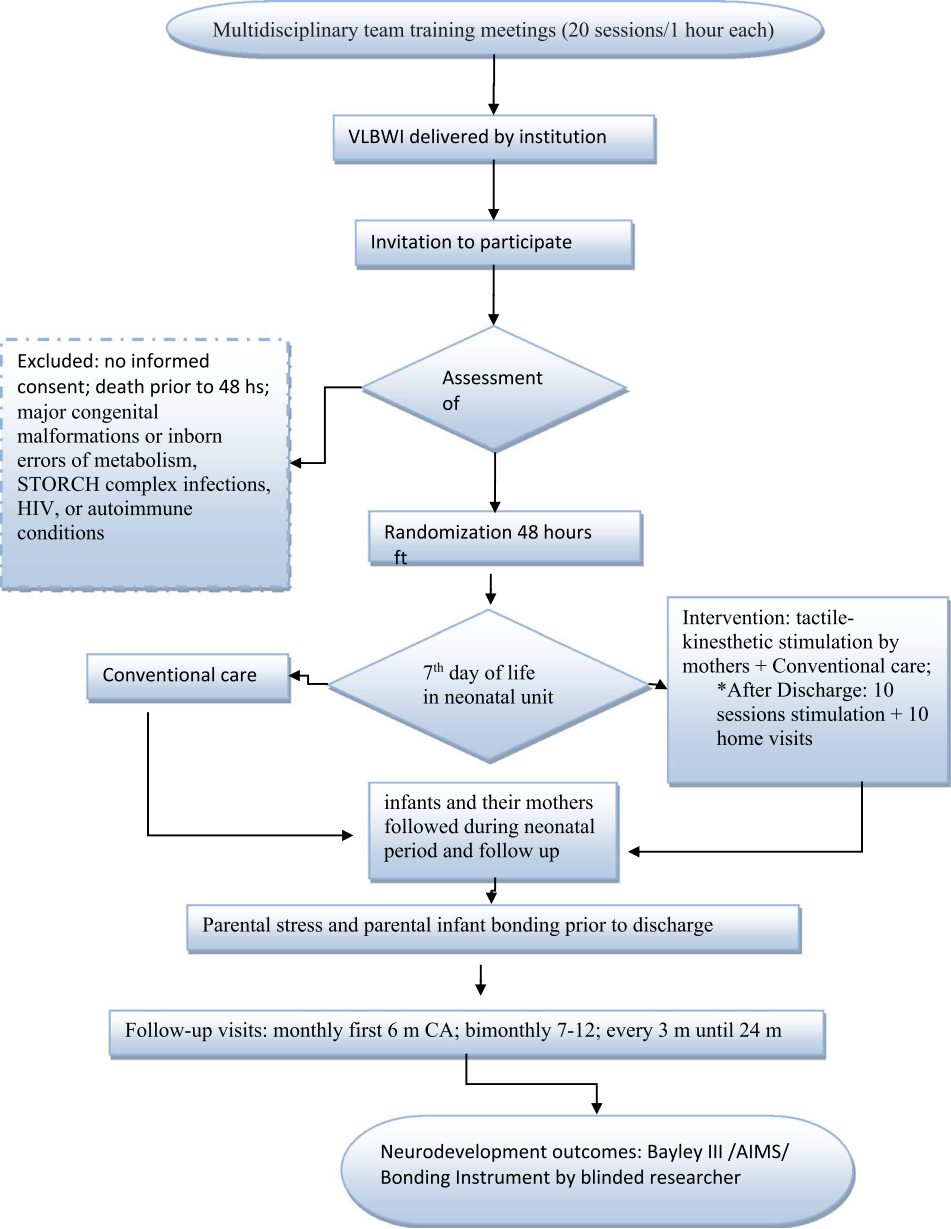
Study flow chart. The flow chart of enrolment, allocation, intervention and assessment. *Intervention Group has ten additional appointments for systematic orientation for a continuous global simulation at home and ten home visits

The study protocol has used the SPIRIT 2013 checklist, so we are presenting the participant timeline [23] (Fig. 1):

1. Multidisciplinary team training meetings (20 sessions/1 h each): All the team will need to be trained to teach the tactile-kinesthetic stimulation by mothers. They are modules of guidelines for the entire multidisciplinary team.
2. Identification of eligible subjects.
3. Written informed consent is read and signed by the parents
4. A researcher have done a randomization method for subjects allocation (48 h after birth)
5. Intervention group (tactile-kinesthetic stimulation by mothers) or conventional care is begun according randomization in the 7th day of life in neonatal unit until hospital discharge.
6. All preterm infants and their mothers are followed during neonatal period.
7. Multidisciplinary team prepares all eligible patients for hospital discharge, promotes regular meeting with the mothers, and high standard guidance for all patients, followed by two groups of care according to the previous randomization.
8. Parental stress and parental infant bonding at hospital discharge in all preterm infants included in the study and survival in the neonatal period is evaluated.
9. All patients are referral to follow up program ten days after discharge.
10. Both arms are referral to monthly follow-up visits until 6 months of corrected age; bimonthly up to 12 months CA; and each 3 months until 24 months CA in both groups.
11. Intervention Group receives ten additional appointments for systematic orientation for a continuous global simulation at home.
12. Following each additional appointment, the multidisciplinary team has does home visits to check and answer mother’s doubts regarding of the stimulation’s program.
13. Neurodevelopment Outcome measure at 12–18 months CA in all patients (both arms) by blinded professional.

Sample size: the sample size was calculated on the basis of the results of many studies that assessed improvement of motor and mental development or cognitive or language acquisitions. All these studies obtained minimum scores 20% higher after early intervention. For a 5% level of significance and a statistical power of 80%, a sample size of 84 patients will be required to detect a 3-point between-group difference in development scores [20, 24–26]. The allocation of the subjects will be perfomed until the complete calculated sample size based on the number of premature infants who survive until hospital discharge. A number of 20% (number 16) will be added, considering possible loss or death during the follow-up. Total sample size will be 100 preterm infants.

Recruitment: strategies for achieving adequate participant enrolment to reach target sample size will be: to establish a link between the research team and mother and father of each of the subjects included, regardless of the group allocated since randomization. Weekly meetings are performed during NICU stay.

A telephone number is available twenty-four for all questions after discharge and during the follow up. This mobile phone stays each day with a researcher’s member on call to resolve doubts with clarity and security. A prior scheduling of home visits and office appointments is done continuously during the recruitment and assessment according the objectives.

Assignment of interventions: Monitoring the progress of this research is fundamental for assuring that all activities will be achieving stated milestones. The selection of adequate method for generating the randomization sequence is important for result measurements. We have used the research randomizer program available at www.randomizer.org [23].

Simple randomization method for allocation: computer-generated random numbers for each 5 participants determines the allocation group. The trial is monitored during the process to have a balance in the number of subjects on each arm over time, eg, twins, triplets; they will be allocated for the same group. We have not stratification by gender, gestational age, or any other variable. In neonatal period, preterm infants will be sequentially randomized when they completed 48 h after birth. Randomization is performed by researchers that will not be responsible for any intervention, nor outcome measures of parental bond, motor and cognitive outcomes. The same researchers will register all neonatal data, discharge variables and the follow-up data, during the appointments (not blinded). During the NICU stay period, the nursing and the medical staff were informed that participating infants would receive an active intervention by the mothers depending on group allocation (not blinded). The data analysts, outcome assessors and the multidisciplinary team working in the institutional follow up clinic will be blinded.

The allocation of the subjects will be maintained until the complete sample size calculation. Both arms are routinely candidates for outpatient institutional follow-up clinic.

Data collection, management and analysis are being presented according checklist [23].

Data collection: Clinical morbidities and identification data were prospectively collected in duplicate during hospital stay until hospital discharge for the two independent researchers.

Both groups have all data obtained during regular appointments at the outpatient follow up clinic masked for allocation of patient groups. All variables will have double data entry in data record center storage.

Neonatal variables include maternal and perinatal characteristics and short-term outcomes. The maternal characteristics are: age, parity, number of prenatal visits, gestational diabetes, chronic hypertension or preeclampsia, chorioamnionitis or urinary tract infection, household income and educational level. The neonatal variables are: gender, type of delivery, 1 and 5 min Apgar scores, surfactant use (at least one dose), antenatal corticosteroid use, gender, gestational age (evaluated by the last menstrual period and confirmed by an early obstetrical ultrasound and neonatal clinical examination), birth weight and small-for-gestational-age status (defined as a birth weight below the 10th percentile), and the Neonatal Acute Physiology and Perinatal Extension II (SNAPPE II) score. The presence of Respiratory Distress Syndrome, bronchopulmonary dysplasia, apnea of prematurity, early or late onset sepsis as confirmed using positive blood cultures, meningitis, necrotizing enterocolitis, patent ductus arteriosus, perintraventricular hemorrhage and periventricular leukomalacia (as determined by brain ultrasound and confirmed using magnetic resonance imaging during the follow-up period within the first 12 months of corrected age), retinopathy of prematurity and universal neonatal hearing screening evaluation (otoacoustic emissions- OAE) during the neonatal period and BERA until six months of corrected age have been also assessed in both arms.

During the follow up three pediatrician/neonatologist will be evaluating monthly, until 6 months of corrected age (CA), bimonthly from 7 up to 12 months corrected age, and every 3 months thereafter until age 24 months, according to routine hospital practice all very low birth weight infants of the study. According randomization conventional group will have standard care of a traditional follow up clinic taking care of the demands according to their necessity and the intervention group, will receive orientation for a continuous global simulation at home besides the usual appointments to the follow up clinic (as previously presented), monthly in the first semester corrected age and bimonthly in the second semester until 12 months corrected age and every 3 months thereafter until age 24 months corrected age. Multidisciplinary team will participate of these activities all the time. All these data can be found in the protocol.

Growth velocity will be registered in the reference curves, using the two computers with an appropriate statistical program for nutritional assessment (ANTHRO). There are two nutritionists to perform the growth evaluations in the exact moments in each group (blinded to which group the child was previously allocated). In the Intervention Group there will be a Nutritionist that will make home visits with the team to reinforce breastfeeding practices. The research team is using a check list during the home visits to the Intervention Group; home visits should be done at least two more researchers.

Statistical methods: All analyses will be performed in the PASW Statistics® for Windows, Version 18.0 software environment (Chicago, IL: SPSS Inc. Released 2009). Qualitative variables will be expressed as absolute and relative frequencies. Pearson chi-square test will be employed to determine the association between categorical variables, with adjusted residuals in case of statistical significance.

Symmetrically distributed continuous variables will be described as means and standard deviations, while asymmetrical distributed categorical variables being described as medians and interquartile ranges. Fisher’s exact test for comparison of categorical variables and Student’s t or the Mann–Whitney tests for comparisons of symmetrically distributed quantitative variables and asymmetrically distributed variables, respectively will be used. Subgroup and any additional analyses will be performed to adjust for social status, maternal age and some preterm’s neonatal conditions associated with poor neurodevelopment outcome.

Monitoring: There are five researchers to monitor data without competing interest. All researchers are independent from sponsor. Data are continuously monitored during the study. The teaching activities and educational programs for the families will be the focus of our research group throughout the study and if some adverse events occur, will be reported. Previous published study showed that the intervention during NICU stay is safe; the 10 additional appointments and 10 home visits will check the intervention safety [23].

Table 2 summarizes the project framework regarding specific objectives, outcomes and period of activities.

**Table 2:** Project Framework: specific objectives, outcomes and period of activities

Specific Objectives	Outcomes	Period acticvities
Objective 1:		
To implement a program of continuous and global intervention for preterm infants to be delivered by their families	Improve interaction parental infants since neonatal period with massage therapy by mothers Decrease parental stress Improve neurodevelopment at 12 to 18 months corrected age	During the whole study period. It start one month after receive the grant. There are meetings with the team during the study. All teamneed to be trained to teach the tactile-kinesthetic stimulation by mothers and home visits. The patients are allocated to the study up to a total of 100 (50 in each group) and sequentially randomized when the patients complete 48 h after birth. Exclusion criteria: congenital malformations and parents’refusing to participate in the study. At the end professionals with training to carry out the testings for development assessments will provide the results.
Objective 2:		
Advise and improve the skills of care givers in respect to children’s needs	Reduce to the minimum the lost for follow up (home visits, phone contacts, phone calls) Decrease parental stress that will be measured previously Improve parental infant bonding that will be measured in the beginning Home visits to be sure that the interventions are performed	Patients are randomized to two groups. At admission of the study the intervention will promote a moment of care and further interaction through massage therapy performed by mothers. Following consist of guidelines and measures to promote early intervention with parents’ orientation independently of f the standard evaluation and care that will be performed. There will be daily sessions of 10 to 5 min each one in NICU and three times a week (motor stimulation), daily (cognitive stimulation) at home.
Objective 3:		
Evaluate the impact of the intervention in the neurodevelopment of the children	More strengthened ties to the program start, as measured by PBI resulting in higher scores of attention, care and protection . Number of patients with the Bayley III scale normal for corrected age Number of patients with AIMS scale normal for corrected age. Statistical measures of the differences between the groups (intervention and conventional approaches).	At discharge of neonatal unit (PBI) we will have the first evaluation. After 10 sessions and home visits we will have the follow up evaluations. AIMS and Bayley III Scales at 12 to 18 months corrected age A global neurodevelopment evaluation will be obtained at 12 to 18 months corrected age for all patients.

### Ethics and dissemination

This study protocol does not involve any harmful procedures or adverse events and it was approved by the Hospital de Clínicas de Porto Alegre (HCPA) Research Ethics Committee (institutional review board-equivalent, judgment number). Written informed consent was obtained from the parents or guardians of all included neonates prior to study enrollment and another two independent informed consent will be present or parents or guardians after randomization, according group allocation. All research data and personal information will be under responsibility of the researchers in order to protect confidentiality before, during and after the trial. All parents or guardians results will be continuously communicated regarding the trial results.

## Discussion

The study protocol is ongoing with a program of early, continuous and global intervention with parents’ orientation independently of the standard evaluation and care that will be performed for preterm infants since NICU stay and after discharge. We have home visits done by the multidisciplinary team to evaluate the comprehension of the orientation and to be sure that the intervention has been done correctly by families. We will provide conditions to establish an early stimulation protocol according to corrected age exercised by the family. Visual, auditory, gross and fine motor skills, socialization, definition and body parts knowledge will be worked out as previously described in this project.

Preterm infants are high risk for delayed neurodevelopment. There are several intervention programs attempting to improve their outcome. Early intervention programs for preterm infants that focus on development while the babies are still in the hospital and post discharge, and into the community setting may have an important impact on long-term morbidity as they are able to focus more on family factors and home environment. Interventions that are aimed at enhancing the parent-infant relationship focus on sensitizing the families to infant’s cues and teach appropriate and timely response to the preterm infant’s needs, possibly that early high-quality parent-infant or mother-infant interactions positively influence cognitive and social development in children [3, 4].

The multidisciplinary approach to early interventions may result in better performance and quality of life in the future for these children. It is known that preterm infants are susceptible to several handicaps like neurological injuries, growth failure, psychiatric problems, visual and hearing deficits, fine and gross motor problems and language problems. A multidisciplinary team is required to form a follow up clinic and measure the outcomes [7, 10, 11]. This clinic must be coordinated by a neonatologist that understands the infant as a whole, as we have proposed in this study protocol [3, 4].

Many studies have been focused only in motor development after early intervention [7]. Recent neuroplasticity literature suggests that intensive, task-specific intervention ought to begin as early as possible and in an enriched environment, during the critical period of neural development. Active motor interventions are effective in some populations. However, the effects of those active motor interventions on the motor outcomes of infants with Cerebral Palsy (CP) have been researched only in a pilot study [24]. Goals - Activity - Motor Enrichment): protocol GAME, was used in that pilot study [25]. The cognition is very poor evaluated after early intervention programs. Recently, the effects of the Teach-Model-Coach-Review instructional approach on caregivers’ use of four enhanced milieu teaching (EMT) language support strategies and on their children’s use of expressive language were examined and the results were positive, but preliminary evaluated [26].

To explore the effect-size of this early intervention program in offer conditions to parents stimulate their preterm children we will provide guidelines for parental bonding. The EAS is a set of scale designed to assess the ability of parents and child to share a healthy emotional connection, therefore addressing the adults and the child relationships [27]. An innnovative and essencial outcome will be evaluate if parents can learn how to support their child’s development of motor and cognitive processes by receiving specialized and multidisciplinary skills training.

The study protocol needs to be share with healthcare professionals in order to use the same approach in other countries with high risk situations for poor neurodevelopment outcome. Thus, if we can show that a continuous and global early intervention at home performed by low income families is better than the standard care for very preterm infants, this kind of program may be applied elsewhere in the world. It can be expanded for the whole preterm population in order to improve their neurodevelopment outcome.

## Abbreviations

AIMS: Alberta Infant Motor Scale
BSDI-III: Bayley Scale Development Index-III edition
CA: correctd age
CG: Conventional Group
CP: cerebral palsy
EAS: Emotional Availability Scale
EMT: enhanced milieu teaching
GA: Gestational Age
GAME: Goals-Activity-Motor-Enrichment
HCPA: Hospital de Clínicas de Porto Alegre
IG: Intervention Group
KMC: Kangaroo Mother Care
NICU: Neonatal Intensive Care Unit
OAE: otoacustic emissions
PBI: Parental Bonding Instrument
RCT: Randomized clinical Trial
SNAPPE II: Score Neonatal Acute Physiology and Perinatal Extension II
STORCH: Syphilis, Toxoplasmosis, Rubella, Citomegalovirus, Herpex virus
WHO: World Health Organization
